# Quality of Life and Anxiety in Age Macular Degeneration Patients: A Cross-Sectional Study

**DOI:** 10.3390/ijerph19020820

**Published:** 2022-01-12

**Authors:** Daniel Caballe-Fontanet, Cristina Alvarez-Peregrina, Neus Busquet-Duran, Eduard Pedemonte-Sarrias, Cristina Andreu-Vázquez, Miguel Ángel Sánchez-Tena

**Affiliations:** 1 Faculty of Biomedical and Health Sciences, Universidad Europea de Madrid, 28670 Madrid, Spain; opticavisiocaballe@gmail.com (D.C.-F.); cristina.andreu@universidadeuropea.es (C.A.-V.); 2 Department of Ophthalmology, Althaia Xarxa Assistencial Universitària de Manresa, 08243 Manresa, Spain; nbusquet@althaia.cat (N.B.-D.); or eduard.pedemonte@umedicina.cat (E.P.-S.); 3 Faculty of Medicine, University of Vic—Central University of Catalonia (UVic—UCC), 08500 Vic, Spain; 4 ISEC LISBOA—Instituto Superior de Educação e Ciências, 1750-179 Lisboa, Portugal; masancheztena@ucm.es; 5 Department of Optometry and Vision, Faculty of Optics and Optometry, Universidad Complutense de Madrid, 28037 Madrid, Spain

**Keywords:** age-related macular degeneration, STAI, NEI VFQ-25, anxiety, quality of life, AMD

## Abstract

(1) Background: Chronic diseases affect patients’ quality of life. Age Macular Degeneration (AMD) is one of the most prevalent chronic ocular diseases. The study aims to measure the anxiety and quality of life related to vision in patients with AMD, as well as the relationship with other visual and demographic parameters. (2) Methods: Prospective cross-sectional study in AMD patients. Visual acuity (VA), contrast sensitivity (CS), line difference in the Colenbrander test, and the degree of pathology were measured. Other variables such as gender, age, and time from the diagnosis were also collected. Anxiety was measured with the Spielberger State-Trait Anxiety Inventory (STAI) and quality of life with the National Eye Institute Visual Function Questionnaire (NEI VFQ-25). The Strengthening the Reporting of Observational Studies in Epidemiology (STROBE) checklist was followed. (3) Results: Patients with higher punctuation in Trait STAI showed lower punctuation in NEI VFQ-25 questionnaire (Spearman coefficient −0.415; *p* = 0.001). The variables VA, CS, and age were correlated to the quality of life. The relationship between trait anxiety and subscales of NEI VFQ-25 was significant for all subscales (*p* < 0.05), except for social functioning, peripheral vision, general vision, ocular pain, and driving. (4) Conclusions: AMD patients with higher levels of anxiety show a decrease in their quality of life. The quality of life of AMD patients depends on their VA and CS.

## 1. Introduction

Age-related Macular Degeneration (AMD) is the leading cause of blindness in the USA and the third cause in the world. The National Eye Institute predicts that 5.4 million people will suffer AMD in the USA by 2050 [1]. As life expectancy increases, the incidence of AMD grows, making AMD an important challenge for Public Health Systems [2].

In a global meta-analysis on almost 130,000 subjects, AMD prevalence was 9% in people between 45- and 85-years old [3]. Prevalence increases with age as the Spanish Eyes Epidemiological Study shows. This study was carried out in different places across Spain, and the authors found an AMD prevalence of 1% in people aged between 65 and 74, and 8% older than 80 years old [4]. A similar prevalence has been found in other places, with a higher prevalence of AMD in Europeans than in Asians and Africans, according to the systematic review and meta-analysis made by Wong et al. in 2014 [5].

AMD has been classified as dry or wet, according to the pathological and clinical signs. The dry or atrophy AMD has a slow development and leads to the degeneration of the retinal pigment epithelium and photoreceptors. Wet or exudative AMD has a quick progression and usually results in a sudden loss of central vision [6].

AMD decreases the patient’s quality of life, similar to what happens in other severe systematic diseases such as cancer, ischemic coronary disease, or ictus. AMD patients have difficulty in making their daily routines, feel they depend on others, suffer depressions, and have a high risk of falling [7].

In a study carried out in Germany in more than 7000 AMD patients aged between 40 and 90, the prevalence of depression and anxiety was related to AMD. Depression and anxiety were more frequently found in AMD patients [8]. In a systematic review carried out by Dawson et al. in 2012, symptoms of depression were more prevalent among AMD populations than anxiety symptoms [9].

According to the World Health Organization, health is a state of complete physical, mental, and social well-being, and not merely the absence of disease or infirmity. Traditionally in ophthalmology, the success of treatment has been measured by morbidity, visual acuity (VA), or the visual field [7]. The development of medicine has resulted in a lot of patients with chronic diseases who live longer, so the quality of life has become an important aspect to study [10,11]. In Optometry and Ophthalmology, some questionnaires to evaluate the quality of life related to vision have been validated in the last years. All of them are self-reported questionnaires that include questions about specific daily activities. Patients were asked to rate the level of difficulty that they experienced, while performing the activity described. Notably, only three of these instruments have been validated on low vision patients, and just two of them have been validated on a wide range of pathologies. One is the VCMI, a 10-item vision-related quality-of-life questionnaire, and the other is the National Eye Institute visual function questionnaire (NEI VFQ), and the shorter version NEI-VFQ-25 [12,13].

Indeed, one of the most extensive questionnaires to measure the quality of life related to vision is the NEI VFQ-25 [14]. It has been translated into multiple languages, such as Spanish [15]. On the other side, the State-Trait Anxiety Inventory (STAI) is one of the most frequently used psychometric tests to value anxiety [16]. It is a validated 20-item self-report assessment device that includes separate measures of state and trait anxiety. The first one is a transient emotional condition, and the second one is an anxious propensity, a latent state. It has also a Spanish adaptation, which was developed in 2011 [17].

The relationship between anxiety, quality of life, and AMD has not been widely studied, so this research aims to correlate results from STAI and NEI-VFQ-25 in patients with AMD.

## 2. Materials and Methods

A prospective cross-sectional study with convenience sampling was carried out. Patients were recruited from Òptica Visió and Althaia Hospital, both in Manresa (Spain). All patients diagnosed with dry AMD and without other ocular pathologies were asked to participate in the study.

The study was conducted according to the guidelines of the Declaration of Helsinki and was approved by the Ethics Committee of “Fundació Unió Catalana Hospitals” (protocol code CEI 18/86 and date of approval 27th November 2018).

The following demographic variables were collected: gender, age, and years from the AMD diagnosed.

Visual variables were also analyzed: VA with the Early Treatment Diabetic Retinopathy Study test (ETDRS) [18], Contrast Sensitivity (CS) using the Pelli–Robson test, line difference in high and low contrast test of Colenbrander (dlTC), and structure (STR) using a 3D OCT Maestro (Topcon, Tokyo, Japan).

All patients completed both the STAI and VFQ-25 questionnaires.

Statistical analysis was conducted using Stata IC v.14 (StataCorp LLC). Qualitative variables were expressed as absolute (n) and relative (%) frequencies. The Shapiro–Wilk test was used to evaluate the parametric behavior of the quantitative variables. Mean values ± standard deviation were given for normal distributions; for non-normal distributions, the data were reported as medians with interquartile range. Pearson’s or Spearman’s correlation analyses were used for correlating anxiety and quality of life. For the NEI VFQ-25 subscales that showed a significant correlation to STAI, multiple linear regressions were performed to evaluate the relationship between the quality of life’s subscale and the following variables: gender, age, years from when the AMD was diagnosed, VA, and CS. For each linear regression, the normality of the residuals was tested, and robust regression models were used in cases of violation of the normality. To assess statistical significance, a cut-off point of *p* < 0.05 was considered.

## 3. Results

The number of enrolled patients in the study was 80. In all, 70% of the subjects were females, and 30% were males. The median value of age was 83.50 years old (IQR 79.00–81.00), and the median value of the time from the diagnosis was 5.00 years (IQR 1.00–8.00). Regarding the severity of the AMD, 15.00% of the subjects were in an early stage, 32.50% in an intermediate state, and 52.50% suffer from an advanced AMD.

The mean values of VA in the subjects were 0.32 ± 0.30 on a LogMAR scale, and the mean values of CS was 1.28 ± 0.30.

Table 1 shows a descriptive analysis of the values found in the variables of STAI and in the quality-of-life questionnaire that did not follow a normal distribution.

The VFQ-25 subscales of near and distance activities followed a normal distribution with mean values of 64.22 ± 26.02 and 60.21 ± 27.20, respectively.

Table 2 shows the correlation between the trait and state anxiety and quality of life in the subject studied. There is no correlation between state anxiety and quality of life. A significant correlation was also found for the trait anxiety and the following subscales: Near activities, distance activities, mental health, role difficulties, dependency, and color vision.

Table 3 shows, through a multiple linear regression model, how the quality of life is correlated to trait anxiety, age, distance VA, and CS.

Table 4, Table 5, Table 6, Table 7, Table 8 and Table 9 show the same data for the subscales of the NEI VFQ-25 questionnaire that have shown a correlation with any of the parameters evaluated. It shows a correlation of all the subscales to VA, and in most of them in CS, age, and years from the diagnosis.

So, Table 4 shows that the subscale “near activities” from the quality-of-life questionnaire is not correlated to trait anxiety; although, there is a correlation between “near activities” and the AMD severity and distance VA.

Table 5 presents how the subscale “distance activities” from the quality-of-life questionnaire is also not related to trait anxiety, finding a correlation between “distance activities” and distance VA.

In Table 6, the correlation between the subscale “mental health” from the NEI-VFQ-25 questionnaire and trait anxiety is shown. The table shows the correlations among “mental health” and age, distance VA, and CS.

Table 7 shows the relationship between the subscale “role difficulties” from the NEI-VFQ-25 questionnaire and trait anxiety. There is a correlation among “role difficulties” and trait anxiety, AMD severity, and distance VA.

Table 8 shows that the subscale “dependency” from the quality-of-life questionnaire is not correlated to trait anxiety; although, there is a correlation between “dependency” and age, distance VA, and CS.

Finally, Table 9 shows the correlation between the subscale “color vision” from the quality-of-life questionnaire and trait anxiety. It shows also the correlation between “color vision” and age, distance VA, and CS.

## 4. Discussion

This study shows how AMD patients suffer from anxiety and have a low quality of life. The decrease in the quality of life is related to the VA, CS, and age. To our knowledge, this is the first study that has considered CS as a parameter related to the anxiety and quality of life of AMD patients.

The aging of the population has meant that chronic and degenerative diseases have increased every year. According to WHO, global life expectancy at birth increased from 66.8 years in 2000 to 73.3 years in 2019 [19]. Although healthy life expectancy has also increased (from 58.3 years to 63.7 years in the same period), due to the aging of population, there are still a lot of people suffering from chronic diseases. There is evidence that young adults with just a single chronic disease have a lower health-related quality of life [20].

Some of the abovementioned chronic diseases are eye diseases, lowering the health-related quality of life. In this sense, Mills et al. reviewed the published studies regarding quality of life in glaucoma and other chronic diseases. They concluded that the impact of glaucoma on quality of life was like the impact of other serious chronic diseases; although, they recommended the use of specific tools to measure the quality of life related to vision for eye pathologies [21].

The National Eye Institute Visual Function Questionnaire (NEI VFQ-25) is the shorter version of the 51-item NEI VFQ. Both are vision-targeted questionnaires that assess the influence of visual impairment caused by different pathologies on quality of life [14].

A meta-analysis carried out by Dawson et al. showed that there is great heterogeneity between the different studies published on the prevalence of anxiety and depression in people with AMD, which makes it difficult to give precise estimates [9]. Depression is a multifactorial pathology, so when it appears in AMD patients, we should consider other pathologies that could be affecting the patient, like ischemic coronary disease or diabetes [8].

The scales of STAI and NEI VFQ-25 questionnaires are inverse. In the first one, a lower score means a lower level of anxiety, while in the second, one lower rate means lower quality of life. In our study, there was a moderate negative correlation between trait anxiety and quality of life, but we do not find any relation between quality of life and state anxiety. Similar results were found by Cingu et al. in keratoconus patients treated with cross-linking, comparing the state before the treatment, and the status after a year [22].

According to our results, a better VA was also related to a higher quality of life and lower levels of trait anxiety in AMD patients. Recent studies using the NEI_VFQ-25 questionnaire have found the same results in terms of quality of life. Roque et al. found that the VA affected six NEI-VFQ-25 subscales: dependence, near activities, role difficulties, mental health, color vision, and composite score [23]. Indeed, other studies have proven that improvements in the VA of AMD patients turn into a better quality of life. Oshima et al. confirmed how VA and quality of life of Japanese AMD patients improved significantly after 12 months of ranibizumab treatment [24]. This result contrasts with a multicenter study carried out in 2007 in in France, Germany, and Italy, where VA was found not to be related to anxiety [25]. This difference is probably due to the differences in the questionnaires used in both studies. Therefore, in our study, patients were obliged to complete the STAI questionnaire, which is considered the Gold Standard, with its strength in measuring state and trait anxiety. In the other study, the patients completed the Hospital Anxiety and Depression Scale, which assesses symptoms experienced during the previous week on a 0–3 scale, so it does not consider the trait anxiety. The differences found highlight the importance of evaluating both trait and state anxiety in chronic diseases. Chronic diseases could have an impact on state anxiety at the beginning of the disease, which could have an impact on trait anxiety with time.

Regarding CS, Nixon et al. found that CS improved in AMD patients after 3 months of treatment with aflibercept, which resulted in an improvement in their quality of life [26]. We cannot compare the relation found between CS, quality of life, and anxiety with other studies because there are no publications that consider this relationship in AMD patients. In our study, CS and VA were correlated to anxiety and quality of life. We already knew that VA decreases with aging, and low contrast VA begins to worsen seven years earlier than high contrast VA [27]. In AMD patients this loss is more severe [28].

The Colenbrander test is used to show one or two lines of difference in subjects without pathologies and regular VA. In our clinical experience, in AMD patients these differences could reach even 10 lines. This test is important to anticipate AMD diagnosis. Changes in the AMD patients’ retinas begin in the perifoveal region, so the contrast difference in this area can anticipate the detection that usually happens when the central region is affected.

Although we did not find a relationship between the years from the diagnosis and anxiety and quality of life, Inna et al. found that anxiety, measured with the Hospital Anxiety Depression Scales (HADS), was positively correlated with duration of e-AMD and patient age, but negatively with vision levels [29]. Again, the use of a different test could give different results; although, the conclusions of the abovementioned study are the same, concluding that AMD patients had anxiety and lower quality of life scores.

Finally, we have found a relationship among most of the subscales of the NEI VFQ-25 and trait anxiety. Depending on the chronicity of the ocular condition, studied levels of anxiety could change. Thus, ocular chronic conditions, such as myopia or pseudo myopia, were associated with anxiety [30,31]. While other ocular pathologies, such as uveitis, showed changes in state anxiety [32] instead of in trait anxiety.

The main limitations of our study are that we did compare to a control group, and the sample was enrolled through a convenience sampling.

## 5. Conclusions

AMD is a chronic and degenerative pathology that severely affects the vision of patients, reducing their quality of life and producing anxiety.

The quality of life of AMD patients depends on the VA and CS.

## Figures and Tables

**Table 1:** Median values of STAI—quality of life in general and in the different subscales in patients suffering.

	Median	Q1	Q3
**Trait Anxiety**	20.00	16.50	24.50
**State Anxiety**	21.50	20.00	23.50
**Quality of Life**	78.21	61.19	88.13
**Subscales of quality of life**			
**General Vision**	40.00	20.00	40.00
**Ocular pain**	100.00	87.50	100.00
**Social function**	87.50	62.50	100.00
**Mental Health**	78.13	56.25	93.75
**Role Difficulties**	75.00	50.00	100.00
**Dependency**	100.00	75.00	100.00
**Driving**	91.67	66.67	95.83
**Peripheral Vision**	100.00	100.00	100.00

**Table 2:** Correlation between state and trait anxiety and the VFQ-25 quality-of-life questionnaire and its subscales.

	State Anxiety		Trait Anxiety	
	Correlation *	*p*-Value	Correlation *	*p*-Value
**Quality of Life**	−0.077	1.000	**−0.415**	**0.001**
**Subscales**				
**General Vision**	−0.002	1.000	−0.269	0.160
**Ocular pain**	−0.005	1.000	−0.147	1.000
**Near Activities**	−0.059	1.000	**−0.319**	**0.040**
**Distance Activities**	0.001	1.000	**−0.327**	**0.031**
**Social function**	−0.035	1.000	−0.212	0.586
**Mental Health**	−0.099	1.000	**−0.424**	**0.001**
**Role Difficulties**	−0.065	1.000	**−0.428**	**0.001**
**Dependency**	−0.172	1.000	**−0.373**	**0.007**
**Driving**	0.044	1.000	−0.246	1.000
**Color Vision**	−0.175	1.000	**−0.356**	**0.013**
**Peripheral Vision**	−0.054	1.000	−0.192	0.875

* Spearman correlation coefficient (Rho). Values in bold means statistical significance.

**Table 3:** Multiple linear regression model that correlates trait anxiety with quality of life.

VFQ-25	Coefficient	SE	*t*	*p* > *t*	(95% CI)	
**Trait Anxiety**	−0.64	0.22	−2.93	**0.005**	−1.07	−0.20
**Age**	0.61	0.17	3.48	**0.001**	0.26	0.95
**Gender (female)**	1.53	2.62	0.59	0.560	−3.69	6.75
**AMD severity**						
**intermediate (vs. early state)**	−3.56	3.68	−0.97	0.337	−10.91	3.79
**Advance (vs. early stage)**	−7.13	3.72	−1.92	0.059	−14.55	0.28
**Years from diagnosis**	0.07	0.21	0.35	0.727	−0.35	0.50
**Distance visual acuity**	33.34	4.71	7.08	**<0.001**	23.95	42.73
**Contrast Sensitivity**	13.89	4.88	2.85	**0.006**	4.15	23.64

SE: Spherical Equivalent; AMD: Age Macular Degeneration; CI: Confidence Interval. Values in bold means statistical significance.

**Table 4:** Multiple linear regression model that correlates trait anxiety with the subscale Near Vision from the quality-of-life questionnaire NEI-VFQ-25.

Near Activities	Coefficient	SE	*t*	*p* > *t*	(95% CI)	
**Trait Anxiety**	−0.36	0.32	−1.12	0.267	−1.00	0.28
**Age**	0.48	0.26	1.87	0.065	−0.03	0.99
**Gender (female)**	2.87	3.85	0.74	0.459	−4.82	10.56
**AMD severity**						
**intermediate (vs. early state)**	−11.75	5.43	−2.17	**0.034**	−22.58	−0.93
**Advance (vs. early stage)**	−21.29	5.47	−3.89	**<0.001**	−32.21	−10.37
**Years from diagnosis**	−0.26	0.31	−0.82	0.415	−0.88	0.37
**Distance visual acuity**	54.20	6.93	7.82	**0.000**	40.36	68.03
**Contrast Sensitivity**	9.68	7.19	1.35	0.183	−4.67	24.04

SE: Spherical Equivalent; AMD: Age Macular Degeneration; CI: Confidence Interval. Values in bold means statistical significance.

**Table 5:** Multiple linear regression model that correlates trait anxiety with the subscale Distance Vision from the quality-of-life questionnaire NEI-VFQ-25.

Distance Activities	Coefficient	SE	*t*	*p* > *t*	(95% CI)	
**Trait Anxiety**	−0.23	0.39	−0.58	0.563	−1.00	0.55
**Age**	0.47	0.31	1.50	0.138	−0.15	1.09
**Gender (female)**	−2.41	4.67	−0.52	0.607	−11.73	6.91
**AMD severity**						
**intermediate (vs. early state)**	−5.66	6.58	−0.86	0.392	−18.78	7.46
**Advance (vs. early stage)**	−12.38	6.63	−1.87	0.066	−25.61	0.85
**Years from diagnosis**	−0.17	0.38	−0.44	0.661	−0.93	0.59
**Distance visual acuity**	58.02	8.40	6.91	**<0.001**	41.26	74.78
**Contrast Sensitivity**	11.26	8.72	1.29	0.201	−6.13	28.65

SE: Spherical Equivalent; AMD: Age Macular Degeneration; CI: Confidence Interval. Values in bold means statistical significance.

**Table 6:** Multiple linear regression model that correlates trait anxiety with the subscale Mental Health from the quality-of-life questionnaire NEI-VFQ-25.

Mental Health	Coefficient	SE	*t*	*p* > *t*	(95% CI)	
**Trait Anxiety**	−1.15	0.41	−2.77	**0.007**	−1.97	−0.32
**Age**	0.90	0.33	2.72	**0.008**	0.24	1.56
**Gender (female)**	1.32	4.97	0.27	0.791	−8.60	11.24
**AMD severity**						
**intermediate (vs. early state)**	−4.95	7.00	−0.71	0.482	−18.91	9.01
**Advance (vs. early stage)**	−9.57	7.06	−1.36	0.180	−23.65	4.51
**Years from diagnosis**	0.48	0.41	1.17	0.245	−0.33	1.28
**Distance visual acuity**	37.44	8.94	4.19	**<0.001**	19.60	55.28
**Contrast Sensitivity**	27.64	9.28	2.98	**0.004**	9.13	46.14

SE: Spherical Equivalent; AMD: Age Macular Degeneration; CI: Confidence Interval. Values in bold means statistical significance.

**Table 7:** Multiple linear regression model that correlates trait anxiety with the subscale Role Difficulties from the quality-of-life questionnaire NEI-VFQ-25.

Role Difficulties	Coefficient	SE	*t*	*p* > *t*	(95% CI)	
**Trait Anxiety**	−0.89	0.43	−2.09	**0.041**	−1.75	−0.04
**Age**	0.29	0.34	0.84	0.401	−0.39	0.97
**Gender (female)**	−2.18	5.15	−0.42	0.673	−12.45	8.08
**AMD severity**						
**intermediate (vs. early state)**	−14.62	7.24	−2.02	**0.047**	−29.07	−0.17
**Advance (vs. early stage)**	−17.19	7.31	−2.35	**0.021**	−31.77	−2.61
**Years from diagnosis**	−0.42	0.42	−1.00	0.319	−1.26	0.42
**Distance visual acuity**	54.47	9.26	5.88	**<0.001**	36.00	72.94
**Contrast Sensitivity**	13.33	9.60	1.39	0.170	−5.83	32.49

SE: Spherical Equivalent; AMD: Age Macular Degeneration; CI: Confidence Interval. Values in bold means statistical significance.

**Table 8:** Multiple linear regression model that correlates trait anxiety with the subscale Role Difficulties from the quality-of-life questionnaire NEI-VFQ-25.

Dependency	Coefficient	SE	*t*	*p* > *t*	(95% CI)	
**Trait Anxiety**	−0.55	0.46	−1.21	0.232	−1.46	0.36
**Age**	1.21	0.37	3.31	**0.001**	0.48	1.94
**Gender (female)**	6.99	5.50	1.27	0.208	−3.99	17.96
**AMD severity**						
**intermediate (vs. early state)**	−2.11	7.75	−0.27	0.786	−17.56	13.34
**Advance (vs. early stage)**	−8.38	7.81	−1.07	0.287	−23.97	7.20
**Years from diagnosis**	0.14	0.45	0.32	0.751	−0.75	1.04
**Distance visual acuity**	36.60	9.90	3.70	<0.001	16.86	56.35
**Contrast Sensitivity**	30.79	10.27	3.00	0.004	10.30	51.28

SE: Spherical Equivalent; AMD: Age Macular Degeneration; CI: Confidence Interval. Values in bold means statistical significance.

**Table 9:** Robust multiple linear regression model that correlates trait anxiety with the subscale Color vision from the quality-of-life questionnaire NEI-VFQ-25.

Color vision	Coefficient	Robust SE	*t*	*p* > *t*	(95% CI)	
**Trait Anxiety**	−1.33	0.27	−4.88	**<0.001**	−1.88	−0.79
**Age**	0.60	0.30	2.02	**0.048**	0.01	1.19
**Gender (female)**	3.75	3.74	1.00	0.321	−3.72	11.22
**AMD severity**						
**intermediate (vs. early state)**	3.65	3.45	1.06	0.295	−3.24	10.53
**Advance (vs. early stage)**	0.53	4.05	0.13	0.896	−7.56	8.62
**Years from diagnosis**	0.57	0.34	1.64	0.106	−0.12	1.27
**Distance visual acuity**	20.81	7.76	2.68	**0.009**	5.33	36.85
**Contrast Sensitivity**	16.92	7.98	2.12	**0.038**	0.99	32.85

SE: Spherical Equivalent; AMD: Age Macular Degeneration; CI: Confidence Interval. Values in bold means statistical significance.
